# Is it time to combine untargeted antifungal strategies to reach the goal of ‘early’ effective treatment?

**DOI:** 10.1186/s13054-016-1404-4

**Published:** 2016-08-12

**Authors:** Andrea Cortegiani, Vincenzo Russotto, Santi Maurizio Raineri, Antonino Giarratano

**Affiliations:** Department of Biopathology and Medical Biotechnologies (DIBIMED), Section of Anesthesia, Analgesia, Intensive Care and Emergency, Policlinico P. Giaccone, University of Palermo, Via del Vespro 129, 90127 Palermo, Italy

A recently published retrospective study by Posteraro et al. [1] investigated the use of (1–3)-β-d-glucan (BDG) as a strategy for antifungal drug administration in patients at high risk of candidemia. The strategy consisted of the administration of antifungals (anidulafungin in most cases) to septic patients with a Candida score ≥ 3 and a positive BDG result (≥80 pg/ml). This untargeted strategy led to better selection of patients, avoiding exposure to antifungals in approximately 73 % of patients with negative BDG results and leading to shortened treatment duration in another 20 % of patients.

Untargeted antifungal treatments (including prophylaxis, pre-emptive and empiric approaches) are the mainstay of early invasive fungal infection (IFI) management [2]. We recently published a Cochrane systematic review investigating the effects of untargeted antifungal treatment in terms of mortality and incidence of IFI in non-neutropenic critically ill patients [3]. Notably, prophylaxis resulted in IFI reduction but it may lead to exposure to antifungals for an unacceptably high proportion of patients, with associated potential adverse effects of antifungals, increased risk of resistance and costs. On the contrary, empiric treatment showed no benefit in terms of IFI reduction and mortality. We hypothesized that this observation may be due to inclusion of patients with a more advanced disease stage [4]. Moreover, many patients receiving antifungals may not need them, leading to the observed lack of benefit. The pre-emptive strategy was less investigated, with only one published randomized controlled study included in the systematic review.

According to the findings of Posteraro et al. [1], a surrogate marker-driven strategy, in association with risk factors, might represent an adequate and cost-effective approach to tailor antifungal treatment to patients who may benefit most. Is it time to abandon classic antifungal treatments to shift towards more pliant ‘early’ antifungal strategies based on risk factors and biomarkers? Data from non-randomized studies suggested that this kind of antifungal strategy might combine advantages of classic treatments with improved selection of patients and reduced exposure to antifungals, also being able to help clinicians to decide when to stop treatments [5]. There is a need for further randomized trials to answer the question of whether surrogate marker/risk factor-based antifungal strategies could be beneficial to our critically ill patients, in comparison with other (old?) untargeted treatments, in terms of efficacy, exposure to antifungals and costs.

## Abbreviations

BDG, beta-d-glucan; IFI, invasive fungal infection
